# The role of genetic predisposition in cardiovascular risk after cancer diagnosis: a matched cohort study of the UK Biobank

**DOI:** 10.1038/s41416-022-01935-y

**Published:** 2022-08-24

**Authors:** Huazhen Yang, Yu Zeng, Wenwen Chen, Yajing Sun, Yao Hu, Zhiye Ying, Junren Wang, Yuanyuan Qu, Fang Fang, Unnur A. Valdimarsdóttir, Huan Song

**Affiliations:** 1 West China Biomedical Big Data Center, West China Hospital, Sichuan University, Chengdu, China; 2 Med-X Center for Informatics, Sichuan University, Chengdu, China; 3 Mental Health Center, West China Hospital of Sichuan University, Chengdu, China; 4 Institute of Environmental Medicine, Karolinska Institutet, Stockholm, Sweden; 5 Center of Public Health Sciences, Faculty of Medicine, University of Iceland, Reykjavík, Iceland; 6 Department of Medical Epidemiology and Biostatistics, Karolinska Institutet, Stockholm, Sweden; 7 Department of Epidemiology, Harvard T.H. Chan School of Public Health, Boston, MA USA

**Keywords:** Epidemiology, Health care

## Abstract

**Background:**

Evidence is scarce regarding the potential modifying role of disease susceptibility on the association between a prior cancer diagnosis and cardiovascular disease (CVD).

**Methods:**

We conducted a matched cohort study of UK Biobank including 78,860 individuals with a cancer diagnosis between January 1997 and January 2020, and 394,300 birth year and sex individually matched unexposed individuals. We used Cox model to assess the subsequent relative risk of CVD, which was further stratified by individual genetic predisposition.

**Results:**

During nearly 23 years of follow-up, an elevated risk of CVD was constantly observed among cancer patients, compared to their matched unexposed individuals. Such excess risk was most pronounced (hazard ratio [HR] = 5.28, 95% confidence interval [CI] 4.90–5.69) within 3 months after a cancer diagnosis, which then decreased rapidly and stabilised for >6 months (HR = 1.22, 95% CI 1.19–1.24). For all the studied time periods, stratification analyses by both levels of polygenic risk score for CVD and by family history of CVD revealed higher estimates among individuals with lower genetic risk predisposition.

**Conclusions:**

Our findings suggest that patients with a recent cancer diagnosis were at an increased risk of multiple types of CVD and the excess CVD risk was higher among individuals with lower genetic susceptibility to CVD, highlighting a general need for enhanced psychological assistance and clinical surveillance of CVD among newly diagnosed cancer patients.

## Introduction

Growing evidence suggests that stressful events, such as natural disasters [1] or the loss of a close relative [2], may lead to an increased risk of cardiovascular disease (CVD). An elevation in the risk of CVD has also been robustly observed among patients with stress-related disorders after a trauma exposure [3, 4]. Moreover, studies have consistently showed an association between a cancer diagnosis and subsequently increased risk of overall or specific subtypes of CVD [5, 6], with possible explanations include that a cancer diagnosis, as a devastating event that usually comes with substantial psychological distress [7–9], can be a significant stressor that may trigger or facilitate the development or clinical presentation of CVD [10–12]. However, as most of previous studies were relied on register-based data and therefore lack of data on environmental and lifestyle factors, these analyses had insufficient control for many important confounders [13]. Also, limited attentions have been paid on the potential differences in immediate- and long-term effects of cancer diagnosis on CVD, rendering difficulties on employment of cost-efficient interventions on CVD prevention.

Genetic factors have been demonstrated to play an important role in the development of CVD [14]. For instance, using data from twins, a Danish study reported more pronounced risk of stroke hospitalisation and stroke death among monozygotic co-twins compared with dizygotic co-twins, indicating that genetic factors increase the risk of stroke [15]. Also, genome-wide association studies (GWAS) have identified many genetic variants (i.e. single-nucleotide polymorphisms [SNPs]), such as rs7212798 within *BCAS3*, rs12122341 near *TSPAN2*, and rs880315 near *CASZ1*, that associated with risk of coronary disease [16], atrial fibrillation [17], and stroke [18], respectively. Therefore, it is plausible that genetic factors could confound or modify the risk of CVD following a cancer diagnosis. However, although several studies have involved family history of CVD as a covariate in their analyses, no attempt has been made to explore whether genetic predisposition to CVD can modify the risk of CVD after a cancer diagnosis, utilising genotyping data.

Therefore, taking advantage of rich information on sociodemographic and behavioural and lifestyle factors, individual-level genotyping data, as well as health-related outcomes, in the UK Biobank, we aimed to examine the associations of cancer diagnosis with incident CVD outcomes. Further, we assessed whether these observed associations can be modified by genetic predisposition to CVD.

## Methods

### Study population

The UK Biobank is a large-scale prospective cohort that recruited over 500,000 participants, aged 40–69 years, from England, Scotland, and Wales during 2006–2010. Information on sociodemographic, lifestyle, and health-related factors was collected at recruitment. Health-related outcomes were obtained by periodical linkage with health and medical records (including primary care and inpatient hospital data, cancer registries, and death registries) from multiple national datasets in England, Scotland, and Wales [19], with the participants’ consent. UK Biobank inpatient hospital data were available for 96% of participants in 1997 and reached full coverage of the UK Biobank population from January 1, 1998 [19]. Data from cancer registries were available for all UK Biobank participants from January 1, 1971 [19]. Death registers recorded deaths of all UK residents since 1855 and therefore have the capability of capturing all deaths for UK Biobank participants during the whole study period [20]. Genotyping data, derived from blood samples collected at baseline [21], were released for approximate 500,000 UK Biobank participants using two closely related arrays (95% shared marker content). The genotyping process for the UK Biobank genotyping data has been described in detail elsewhere [21].

**Fig. 1:**
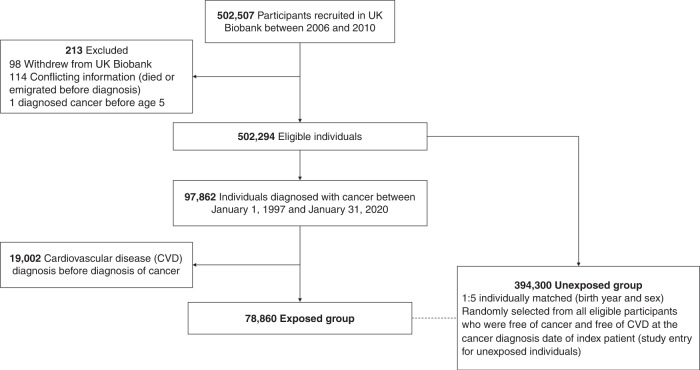
Study design.

In the present study, we conducted a matched cohort study using data from 502,507 UK Biobank participants (Fig. 1). We first excluded individuals who had withdrawn from the UK Biobank (*N* = 98), had conflicting information (died or emigrated before the diagnosis of cancer; *N* = 114), and had their cancer diagnosis before age 5 (*N* = 1), leaving 502,294 eligible participants for further analyses. Individuals with a first cancer diagnosis between January 1, 1997 and January 31, 2020 who had no history of CVD, defined as the status of CVD diagnosis in self-reported, primary care, or hospital inpatient data, were included in the exposed group (*N* = 78,860). For each exposed cancer patient (the index patient), we randomly selected five unexposed individuals, individually matched by birth year (±5 years) and sex, from all eligible participants who were free of cancer and CVD at the cancer diagnosis date of the index patient (i.e. the index date for both exposed and matched unexposed individuals).

All study participants were followed from the index date until a first diagnosis of CVD (any CVD or specific subtype of CVD), death, emigration, or the end of follow-up (January 31, 2020), whichever occurred first. The follow-up of the matched unexposed individuals was additionally censored at the time of their diagnosis of cancer, if any, during the follow-up.

All the UK Biobank participants gave written informed consent before data collection. The UK Biobank has full ethical approval from the NHS National Research Ethics Service (16/NW/0274), and this study was approved by the biomedical research ethics committee of West China Hospital (2019.1171).

### Ascertainment of cancer

We identified a cancer diagnosis between January 1, 1997 and January 31, 2020 based on records from cancer registries or a hospital admission with a diagnosis of cancer in the UK Biobank inpatient hospital data, according to the International Classification of Diseases (ICD) 10th edition (ICD-10) codes C00-C97. The cancer register in UK has been demonstrated high completeness (98–100%), which however can take up to 5 years after a given calendar date to reach full coverage [22]. In sub-analysis, we separately analysed prostate cancer, breast cancer, colorectal cancer, skin cancer, lymphatic or haematopoietic cancer, lung cancer, severe cancer (i.e., oesophageal, liver, or pancreatic cancer), and other cancer, according to the ICD-10 codes (Supplementary Table 1) [12].

### Ascertainment of CVD

Based on UK Biobank inpatient hospital data and mortality data, we defined an incident CVD event (any or specific subtypes including ischaemic heart disease, cerebrovascular disease, emboli/thrombosis, heart failure, and arrhythmia/conduction disorder) as any hospital admission with a diagnosis of CVD or as a death with CVD as the underlying cause, using corresponding ICD-10 codes (Supplementary Table 1). We further defined acute CVD events (i.e. cardiac arrest, acute myocardial infarction, and acute cerebrovascular disease, Supplementary Table 1) as a group of immediate cardiac consequences that could be theoretically triggered by severe stress reactions [3] and unlikely to be affected by the ascertainment bias (i.e. cancer patients were more likely receiving a CVD diagnosis duo to their frequent contacts with medical care systems than non-cancer individuals). CVD death was defined as a death with CVD as the underlying cause.

### Genetic predisposition to CVD

In the present study, we measured the genetic risk of CVD in two ways. First, we used polygenic risk score (PRS) as a proxy of individual’s genetic susceptibility to CVD. Briefly, after performing a standard GWAS quality control [23] for the available imputed genotypes (Supplementary Fig. 1), we included genotyping data from 376,833 participants in the PRS calculation. Based on the summary statistics of GWAS on coronary artery disease from the CARDIoGRAMplusC4D Consortium (i.e. as the base dataset for risk allele weighting, see Supplementary Table 2 for details) that included 60,801 cases and 123,504 controls from 48 studies in 8 countries [16], PRS was computed using penalised regression (LASSO) [24]. In a validation step, the PRS for CVD showed a strong association with the CVD phenotypes in our dataset, measured by logistic regression model adjusted for birth year, sex, genotyping batch, and the first ten principal components for population heterogeneity (odds ratios = 1.21, 95% confidence interval (CI) [1.21–1.23] for a unit increase in PRS).

We then considered family history of CVD (i.e. familial predisposition to CVD) as the alternative indicator for individuals’ genetic predisposition to CVD. Family history of CVD was defined by the self-reported history of heart disease or stroke among first-degree relatives (including father, mother, and siblings) at baseline.

### Covariates

Information about potential confounders, including birth year, sex, race/ethnicity, educational attainment, and behavioural and lifestyle factors (i.e., smoking, alcohol use, physical activity, and dietary intake) was collected at baseline through questionnaires. Diet types were defined as vegetarians, fish eaters, fish and poultry eaters, and meat-eaters based on the collected dietary intake [25]. Data on anthropometry (e.g. height and weight) were measured at the assessment centres at baseline. Body mass index (BMI) was calculated as weight in kilograms (kg) divided by the square of height in metres (m^2^). Townsend deprivation index, a measure of area-level socioeconomic deprivation [26], was assigned to each participant according to the postcode of their address. History of psychiatric disorders (ICD-10 codes: F00-F99) and somatic diseases (i.e. hypertension and diseases considered influential for survival time, including chronic pulmonary disease, connective tissue disease, diabetes, HIV infection/AIDS, liver disease, renal disease, and ulcer disease [27], ICD-10 codes listed in Supplementary Table 1) was defined as a diagnosis of these diseases according to UK Biobank self-reported, primary care, and inpatient hospital data, before the index date. Based on UK Biobank inpatient hospital data, we obtained information on chemoradiotherapy for all cancer patients, using corresponding ICD-10 codes (Supplementary Table 1). All missing values of the covariates were coded to “unknown” category.

### Statistical analysis

Because the elevation of CVD risk after a cancer diagnosis may be time-dependent [28], we first visualised the association between cancer diagnosis and incident CVD events by time since the index date (i.e. the cancer diagnosis date of the index patient), using flexible parametric survival models. Correspondingly, we assessed the relative risk of CVD in relation to cancer diagnosis, using hazard ratios (HRs) with 95% CIs derived from Cox regression models, separately for <3 months, 3–6 months, or >6 months of follow-up. The models were stratified by the matching variables (i.e., birth year and sex), and partly (models 1–4) or fully (model 5) adjusted for birth year (as a continuous variable), race/ethnicity (White or others), the Townsend deprivation index (as a continuous variable), college/university degree (yes, no, or unknown), BMI (<18.5, 18.5–24.9, 25.0–29.9, ≥30.0 kg/m^2^, or unknown), alcohol use (never, ever, or unknown), smoking (never, ever, or unknown), physical activity (low, moderate, high, or unknown), diet types (vegetarians, fish eaters, fish and poultry eaters, meat-eaters, or unknown), history of psychiatric disorders (yes or no), history of somatic diseases (yes or no), and family history of CVD (yes or no). In addition to considering all CVDs as a group, we also examined the specific types of CVD (i.e. ischaemic heart disease, cerebrovascular disease, emboli/thrombosis, heart failure, arrhythmia/conduction disorder, acute CVDs, and CVD death).

To study the role of genetic predisposition in the association between cancer diagnosis and CVD risk, we stratified the analyses by PRS of CVD and performed separate analyses for individuals with a low (<first tertile of PRS), intermediate (between first and second tertile), and high (>second tertile) genetic risk of CVD. Similarly, we did stratification analysis by family history of CVD. When studying subtypes of cancer, the time periods were reclassified as ≤6 months and >6 months of follow-up, to maintain sufficient statistic power.

In stratification analyses, HRs were calculated separately by age at index time (by tertiles: ≤58, 59–65, or ≥66 years), sex, history of psychiatric disorders, and history of somatic diseases. The statistical significance of difference between HRs was assessed by including an interaction term in the Cox model or by Wald test. The impact of chemoradiotherapy on the studied associations was detected by sub-grouping the cancer patients according to the status of chemoradiotherapy. Further, to examine the role of genetic susceptibility to anxiety or stress-related disorders, as an indicator of inherent vulnerability to psychological stress, on the studied associations, we conducted a stratification analysis by the PRS for anxiety- or stress-related disorder (<first tertile, first–second tertile, or >second tertile), calculated based on the GWAS summary statistics of independent samples [29].

To test the robustness of the observed associations to the definition of CVD, we repeated the analyses using merely the primary diagnosis in UK Biobank inpatient hospital data for CVD ascertainment. To further release the concern of ascertainment bias, we calculated the number of hospital admissions during the first year of follow-up and conducted sensitive analysis by additionally adjusting for or stratifying by this variable in the Cox models. In addition, given most lifestyle-related factors were collected at recruitment (i.e. 2006–2010) that might not accurately reflect the conditions at the time of cancer diagnosis, we repeated the main analyses by restricting to participants with the index date right after the baseline data collection (i.e. 1 year after the baseline; *N* = 23,178). All the analyses were done with the R software (version 4.0) and PLINK (version 1.9). A two-sided *p* < 0.05 was considered statistically significant.

## Results

Of the 502,294 eligible UK Biobank participants, we included 78,860 exposed individuals with a diagnosis of cancer (93.29% ascertained by cancer register and 6.71% by hospital inpatient data only, with no differences regarding basic characteristics [Supplementary Table 3], and their 394,300 birth year- and sex-matched unexposed individuals in the matched cohort (Fig. 1). With a total of 3,652,774 accumulated person-years, the mean follow-up time was 7.63 (SD 6.05) and 7.74 (SD 5.71) years for exposed patients and matched unexposed individuals, respectively (Table 1). The mean age at index date was 61.60 years (SD 8.74) and 46.13% study participants were male. While there was no difference in sociodemographic factors and history of psychiatric disorders, patients with a cancer diagnosis tended to be ever smoker (47.73 vs 45.59%), had a higher prevalence of somatic diseases (23.02 vs 21.34%), but lower possibility of having family history of CVD (57.92 vs 59.18%), compared with the unexposed individuals.

**Table 1:** Characteristics of the study cohort.

	Exposed patients	Matched unexposed individuals	Overall
	(*N* = 78,860)	(*N* = 394,300)	(*N* = 473,160)
Birth year, mean (SD)	1950 (7.09)	1950 (6.86)	1950 (6.90)
Follow-up time, mean (SD), years	7.63 (6.05)	7.74 (5.71)	7.72 (5.77)
Age at the index date, mean (SD), years	62.00 (8.88)	61.60 (8.71)	61.60 (8.74)
Age at the index date, no. (%), years			
≤58	25,300 (32.08%)	132,416 (33.58%)	157,716 (33.33%)
59–65	25,783 (32.69%)	131,885 (33.45%)	157,668 (33.32%)
≥66	27,777 (35.22%)	129,999 (32.97%)	157,776 (33.35%)
Sex, no. (%)			
Female	42,478 (53.87%)	212,390 (53.87%)	254,868 (53.87%)
Male	36,382 (46.13%)	181,910 (46.13%)	218,292 (46.13%)
Race/ethnicity, no. (%)			
White	76,297 (96.75%)	374,547 (94.99%)	450,844 (95.28%)
Others	2563 (3.25%)	19,753 (5.01%)	22,316 (4.72%)
Townsend deprivation index, mean (SD)	−1.56 (2.97)	−1.47 (3.00)	−1.48 (3.00)
College or University degree, no. (%)			
Yes	24,539 (31.12%)	123,085 (31.22%)	147,624 (31.20%)
No	37,689 (47.79%)	189,036 (47.94%)	226,725 (47.92%)
Unknown	16,632 (21.09%)	82,179 (20.84%)	98,811 (20.88%)
Body mass index, no. (%), kg/m^2^			
<18.5	398 (0.50%)	1894 (0.48%)	2292 (0.48%)
18.5–24.9	25,792 (32.71%)	126,804 (32.16%)	152,596 (32.25%)
25.0–29.9	34,258 (43.44%)	170,878 (43.34%)	205,136 (43.35%)
≥30.0	18,061 (22.90%)	92,594 (23.48%)	110,655 (23.39%)
Unknown	351 (0.45%)	2130 (0.54%)	2481 (0.52%)
Smoking status, no. (%)			
Never	40,786 (51.72%)	212,239 (53.83%)	253,025 (53.48%)
Ever	37,643 (47.73%)	179,771 (45.59%)	217,414 (45.95%)
Unknown	431 (0.55%)	2290 (0.58%)	2721 (0.58%)
Alcohol status, no. (%)			
Never	2905 (3.68%)	16,795 (4.26%)	19,700 (4.16%)
Ever	75,758 (96.07%)	376,362 (95.45%)	452,120 (95.55%)
Unknown	197 (0.25%)	1143 (0.29%)	1340 (0.28%)
Diet types, no. (%)			
Vegetarians	1071 (1.36%)	6808 (1.73%)	7879 (1.67%)
Fish eaters	1702 (2.16%)	8941 (2.27%)	10,643 (2.25%)
Fish and poultry eaters	1932 (2.45%)	9807 (2.49%)	11,739 (2.48%)
Meat-eaters	74,060 (93.91%)	368,110 (93.36%)	442,170 (93.45%)
Unknown	95 (0.12%)	634 (0.16%)	729 (0.15%)
Physical activity, no. (%)			
Low	11,767 (14.92%)	56,857 (14.42%)	68,624 (14.50%)
Moderate	26,097 (33.09%)	130,726 (33.15%)	156,823 (33.14%)
High	25,204 (31.96%)	127,972 (32.46%)	153,176 (32.37%)
Unknown	15,792 (20.03%)	78,745 (19.97%)	94,537 (19.98%)
History of psychiatric disorders, no. (%)			
Yes	13,281 (16.84%)	62,098 (15.75%)	75,379 (15.93%)
No	65,579 (83.16%)	332,202 (84.25%)	397,781 (84.07%)
History of somatic diseases^a^, no. (%)			
Yes	18,150 (23.02%)	84,138 (21.34%)	102,288 (21.62%)
No	60,710 (76.98%)	310,162 (78.66%)	370,872 (78.38%)
CVD polygenic risk score, no. (%)			
Low	20,934 (26.55%)	99,611 (25.26%)	120,545 (25.48%)
Intermediate	20,173 (25.58%)	100,374 (25.46%)	120,547 (25.48%)
High	19,928 (25.27%)	100,654 (25.53%)	120,582 (25.48%)
Unknown	17,825 (22.60%)	93,661 (23.75%)	111,486 (23.56%)
CVD family history, no. (%)			
Yes	45,672 (57.92%)	233,364 (59.18%)	279,036 (58.97%)
No	33,188 (42.08%)	160,936 (40.82%)	194,124 (41.03%)
CVD outcome, no. (%)			
Yes	13,602 (17.25%)	48,729 (12.36%)	62,331 (13.17%)
No	65,258 (82.75%)	345,571 (87.64%)	410,829 (86.83%)

^a^History of chronic pulmonary disease, connective tissue disease, diabetes, renal disease, liver disease, ulcer disease, HIV infection/AIDS, and hypertension.

During the nearly 23 years of follow-up, 13,602 CVD events were observed among the exposed patients (crude incidence rate, 22.60 per 1000 person-years) and 48,729 among the unexposed individuals (15.97 per 1000 person-years). We observed a peak of CVD risk immediately after cancer diagnosis, followed by a rapid decline within the first three months of follow-up (Supplementary Fig. 2). From 6 months after cancer diagnosis onward, the magnitude of the HRs tended to be stabilised. Estimates obtained from Cox models by follow-up periods showed similar result pattern (Table 2). The fully adjusted HRs (model 5) were 5.28 (95% CI 4.90–5.69), 3.17 (95% CI 2.90–3.47), and 1.22 (95% CI 1.19–1.24) for <3, 3–6, and >6 months of follow-up, respectively. We found increased risk for all the studied subtypes of CVD among the exposed patients (Table 2), with the greatest HRs observed for emboli/thrombosis, heart failure, and cerebrovascular disease.

**Table 2:** Risk of cardiovascular disease (CVD) among patients with a diagnosis of cancer, compared with their matched unexposed individuals.

	<3 months of follow-up		3–6 months of follow-up		>6 months of follow-up	
	No. of events (incidence rate^e^) in cancer patients/matched unexposed individuals	Hazard ratio (95% confidence interval)	No. of events (incidence rate^e^) in cancer patients/matched unexposed individuals	Hazard ratio (95% confidence interval)	No. of events (incidence rate^e^) in cancer patients/matched unexposed individuals	Hazard ratio (95% confidence interval)
*Any CVD*						
Different adjustment strategies^a^						
* Model 1*: adjusted for birth year	1512 (78.56)/1416 (14.50)	5.27 (4.90–5.67)	858 (46.64)/1380 (14.47)	3.15 (2.89–3.44)	11,232 (19.90)/45,933 (16.07)	1.20 (1.17–1.22)
* Model 2*: Model 1 + sociodemographic factors^b^		5.29 (4.91–5.69)		3.18 (2.91–3.47)		1.21 (1.19–1.24)
* Model 3*: Model 2 + behavioural and lifestyle factors^c^		5.24 (4.86–5.64)		3.19 (2.92–3.49)		1.22 (1.19–1.24)
* Model 4*: Model 3 + history of psychiatric disorders+ history of somatic disease		5.24 (4.86–5.64)		3.15 (2.89–3.45)		1.21 (1.19–1.24)
* Model 5 (full model)*: Model 4 + family history of CVD		5.28 (4.90–5.69)		3.17 (2.90–3.47)		1.22 (1.19–1.24)
*Subtypes of CVD*^d^						
Ischaemic heart disease	242 (12.52)/593 (6.07)	2.01 (1.72–2.34)	159 (8.56)/632 (6.62)	1.30 (1.08–1.55)	4175 (7.22)/20718 (7.14)	0.98 (0.95–1.01)
Cerebrovascular disease	126 (6.52)/154 (1.58)	4.09 (3.17–5.27)	86 (4.63)/184 (1.93)	2.31 (1.76–3.04)	2050 (3.51)/7972 (2.72)	1.24 (1.17–1.30)
Emboli/thrombosis	322 (16.67)/74 (0.76)	23.96 (17.93–32.01)	260 (14.01)/76 (0.8)	18.30 (13.80-24.27)	1551 (2.65)/2881 (0.98)	2.81 (2.63–3.00)
Heart failure	96 (4.97)/96 (0.98)	5.55 (4.02–7.67)	59 (3.17)/86 (0.90)	3.51 (2.44–5.06)	1554 (2.66)/5711 (1.95)	1.32 (1.24–1.40)
Arrhythmia/conduction disorder	624 (32.31)/530 (5.42)	5.72 (5.07–6.45)	324 (17.48)/506 (5.30)	3.26 (2.82–3.77)	5277 (9.09)/21,148 (7.24)	1.20 (1.16–1.24)
*Acute CVD*	159 (8.22)/275 (2.81)	2.82 (2.30–3.45)	107 (5.76)/287 (3.00)	1.90 (1.51–2.40)	2552 (4.37)/10,734 (3.66)	1.16 (1.11–1.22)
*CVD death*	17 (0.88)/29 (0.30)	2.84 (1.56–5.17)	15 (0.80)/35 (0.37)	2.10 (1.14–3.84)	516 (0.84)/2185 (0.71)	1.07 (0.97–1.19)

^a^Cox model was used to estimate hazard ratios (HRs), stratified by the matching variables (i.e. birth year and sex), and adjusted for covariates listed in the model information column.

^b^Race/ethnicity, Townsend deprivation index, and educational attainment.

^c^Body mass index, alcohol status, smoking status, physical activity, and diet types.

^d^Cox model was used to estimate hazard ratios (HRs), stratified by the matching variables (i.e. birth year and sex), and adjusted for birth year, race/ethnicity, Townsend deprivation index, educational attainment, body mass index, alcohol status, smoking status, physical activity, diet types, history of psychiatric disorders, history of somatic disease, and family history of CVD.

^e^Number of cases per 1000 person-years.

We found that the observed CVD risk elevations seemed to be higher among individual with low genetic (i.e. <first tertile PRS for CVD) or familial (i.e. no family history of CVD) predisposition to CVD (Table 3). For instance, with the first 3 months of follow-up, the HR was 7.71 [95% CI 5.86–10.14] and 4.26 [95% CI 3.38–5.38] for individuals with low and high PRS, respectively (*p* for difference = 0.0012); and the corresponding HRs for >6 months were 1.26 [95% CI 1.19–1.35] and 1.15 [95% CI 1.08–1.22] (*p* for difference = 0.039). Likewise, the differential risk patterns were also noted when stratifying by CVD family history.

**Table 3:** Risk of cardiovascular disease (CVD) among patients with a diagnosis of cancer, compared with their matched unexposed individuals, by different genetic risk of CVD.

Characteristics	<3 months of follow-up		3–6 months of follow-up		>6 months of follow-up	
	No. of CVD (incidence rate^d^) in cancer patients/matched unexposed individuals	Hazard ratio (95% confidence interval)	No. of CVD (incidence rate^d^) in cancer patients/matched unexposed individuals	Hazard ratio (95% confidence interval)	No. of CVD (incidence rate^d^) in cancer patients/matched unexposed individuals	Hazard ratio (95% confidence interval)
By CVD polygenic risk score, by tertiles^a^						
Low	423 (82.83)/309 (12.52)	7.71 (5.86–10.14)	229 (47.00)/294 (12.19)	3.93 (2.95–5.25)	2864 (19.07)/10,818 (14.95)	1.26 (1.19–1.35)
Intermediate	416 (84.52)/405 (16.30)	5.36 (4.24–6.77)	238 (50.60)/342 (14.10)	4.03 (3.01–5.39)	2944 (20.58)/12,371 (17.09)	1.17 (1.10–1.24)
High	399 (82.1)/470 (18.86)	4.26 (3.38–5.38)	249 (53.65)/482 (19.83)	2.41 (1.88–3.09)	3360 (23.69)/14,328 (19.78)	1.15 (1.08–1.22)
* p*_difference_^b^		0.0012		0.012		0.039
By CVD family history^c^						
No	648 (80.10)/490 (12.30)	6.52 (5.51–7.71)	326 (42.19)/461 (11.85)	3.61 (2.96–4.39)	4329 (18.41)/16,345 (14.06)	1.30 (1.25–1.36)
Yes	864 (77.45)/926 (16.02)	4.89 (4.36–5.48)	532 (49.86)/919 (16.28)	3.11 (2.74–3.54)	6903 (20.97)/29,588 (17.45)	1.17 (1.14–1.21)
* p*_difference_		0.0055		0.21		0.00013

^a^Cox model was used to estimate hazard ratios (HRs), stratified by the matching variables (i.e. birth year and sex) and adjusted for birth year, Townsend deprivation index, educational attainment, body mass index, alcohol status, smoking status, physical activity, diet types, history of psychiatric disorders, and history of somatic disease.

^b^The differences in hazard ratios for CVD polygenic risk score were assessed between low and high subgroups by Wald test.

^c^Cox model was used to estimate hazard ratios (HRs), stratified by the matching variables (i.e. birth year and sex), and adjusted for birth year, race/ethnicity, Townsend deprivation index, educational attainment, body mass index, alcohol status, smoking status, physical activity, diet types, history of psychiatric disorders, and history of somatic disease.

^d^Number of cases per 1000 person-years.

**Fig. 2:**
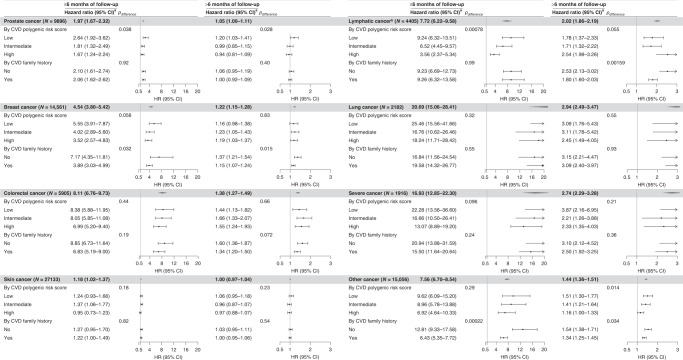
Risk of cardiovascular disease (CVD) among patients with different cancer diagnosis, compared with their matched unexposed individuals, by different genetic risk of CVD. The differences in hazard ratios for CVD polygenic risk score were assessed between low and high subgroups by Wald test. ^a^Cox model was used to estimate hazard ratios (HRs), stratified by the matching variables (i.e. birth year and sex), and adjusted for birth year, race/ethnicity, Townsend deprivation index, educational attainment, body mass index, alcohol status, smoking status, physical activity, diet types, history of psychiatric disorders, history of somatic disease, and family history of CVD. ^b^Lymphatic or haematopoietic cancer.

The increased CVD risk was consistently observed for all studied subtypes of cancer, with the highest HRs observed for severe cancer and lung cancer. By level of PRS for CVD, we again observed slightly higher estimates in the groups with low genetic predisposition to CVD (Fig. 2 and Supplementary Table 4). Our further stratification analyses revealed that the observed associations did not differ by pre-existed psychiatric disorders and history of somatic diseases (Supplementary Table 5) but seemed stronger among younger or female individuals. Although the estimates were higher among cancer patients who received chemoradiotherapy, we still observed significantly increased CVD risk among cancer patients without a chemoradiotherapy (Supplementary Table 6). In addition, we observed enhanced magnitude of the studied associations among individuals with higher genetic predisposition to anxiety or stress-related disorders, particularly for the period within 3 months of follow-up (*p* for difference = 0.033, Supplementary Table 7).

In sensitivity analyses, we obtained largely comparable results when focusing on CVDs identified by only primary diagnosis of inpatient hospital data (Supplementary Table 8), and when restricting the analyses to participants with the index date 1 year after the baseline information collection (Supplementary Table 9). Furthermore, both additionally adjusting for the number of hospital admissions during the first year of follow-up and stratifying by this variable led to slightly lower estimates, with however largely identical risk patterns as the main analyses (Supplementary Table 10).

## Discussion

In this large community-based cohort in the UK, we found that individuals with a cancer diagnosis were at an increased risk of multiple types of CVDs after adjusting for many confounders. The risk increase was greatest during the period adjacent to the cancer diagnosis (i.e. within the first 3 months) but was significant for the whole study period (nearly 23 years of follow-up). In addition, both short- and long-term excess risk of CVD tended to be more pronounced among individuals with low genetic or familial predisposition to CVD, highlighting an importance of environmental factors, including psychological stress induced by a cancer diagnosis, on CVD development particularly among individuals that conventionally considered having low CVD risks (e.g. without family history of CVD). Such findings further motivate timely psychological assistance and enhanced clinical surveillance for CVD, especially acute CVD events, among recently diagnosed cancer patients, irrespective of their disease susceptibility to CVD.

Our finding of an increased CVD risk after a cancer diagnosis is consistent with our previous work, suggesting cancer diagnosis as a stressor that can lead to severe cardiovascular consequences [10–12]. Due to the observational nature of these studies, a concern of residual confounding including genetic predisposition to CVD remains. Further evidence supporting a link between cancer diagnosis and CVDs include studies exploring the relationship of these two traits in the perspective of shared genetic aetiology [30]. However, the approach of identifying a high-risk group of cancer patients in high demand of CVD prevention remains largely unexplored. In our study, we, for the first time, provided a thorough assessment on the influence of genetic predisposition to CVD, indexed by both PRS and family history of CVD, on the association between cancer diagnosis and the risk of subsequent CVD events, controlling for many important confounders. Importantly, as higher risk estimates were noted among cancer patient with lower genetic or familial predisposition to CVD, our results indicated that the environmental factors, such as traumatic life events, might be more influential, in terms of promoting or triggering the development of CVD, among individuals with low disease susceptibility to CVD. Indeed, a further enhance magnitude of association between stress-related disorders on subsequent CVD consequence was reported among individuals without family history of CVD in our previous study based on nationwide register data in Sweden [3].

The underlying mechanisms for the association between cancer diagnosis and CVD remain inconclusive. Possible explanations include the overactivation of the hypothalamic–pituitary–adrenal axes under the conditions of severe stress response [31, 32], which might have direct impacts on cardiovascular system, presenting as increased blood pressure and vascular tone [33]. Also, the autonomic dysfunction [34], endothelial damage [35], and behaviour-related changes [36] that observed among individuals experiencing stressful events might alter CVD risk, both in short (e.g. through precipitating dysrhythmia or left-ventricular dysfunction) and long (e.g. by accelerating the atherosclerotic process) term [32, 37, 38]. In the present study, this psychological stress-related notion was supported by our further finding of further increased excess risk of CVD among individuals with high genetic susceptibility to anxious or stress-related disorder.

In addition to the psychological stress induced by a cancer diagnosis, shared risk factors [39], cancer biology including inflammation [40], and side effect of certain cancer treatment [6] may be alternative explanations for the increased risk of CVD observed among cancer patients. Nevertheless, we observed a distinct high risk of CVD immediately after the cancer diagnosis, after controlling for important confounders (e.g., smoking, BMI, physical activity, and history of somatic diseases), which consistently sustained among cancer patients with and without a chemoradiotherapy, suggesting that neither shared risk factors nor cancer treatment can fully explain the observed results. Also, the excess CVD risk was more prominent after a diagnosis of cancer with poorer prognosis (i.e. severe cancer, including oesophageal, liver, or pancreatic cancer), which may serve as severe life stressors and evoke severe stress reaction [41]. Both phenomena support the notion that psychological stress plays a major role in the elevated CVD risk shortly following a cancer diagnosis. The long-term effect of cancer diagnosis on CVD risk, however, might attribute to many factors, including behaviour-related changes or cancer treatment. Therefore, the mechanism on prolonged CVD risk after a cancer diagnosis deserve further investigation.

The major merits of our study include the prospective design and the large sample size, which enabled the assessment on the associations between different cancers and multiple types of CVD in detail while controlling for important behavioural and lifestyle confounders. Furthermore, taking advantage of the available individual-level genotype data, we applied two ways to measure the genetic predisposition to CVD, i.e. PRS and self-reported family history. Last, using the data from the baseline questionnaires and linkages to health records, we were able to consider a wide range of important confounders, including sociodemographic and lifestyle factors and various psychiatric disorders and somatic diseases, in our analyses.

Notable limitations of this study include the varying accuracy of CVD diagnoses in the UK hospital inpatient data, which was high for stroke (positive predictive value >90%) but less so for coronary heart disease (72%) [42, 43]. Second, the differential surveillance levels between exposed and unexposed groups (i.e. ascertainment bias) could be a concern. Nevertheless, as the analyses specifically focusing on acute and severe CVDs and those further accounting for the number of hospital admissions during the first year of follow-up found slightly low but similar estimates, it is unlikely that such a bias can fully explained the observed associations. Third, the publicly available summary statistics of GWAS used for PRS calculation were mainly generated for coronary artery disease, whereas we studied more types of CVD in the present study. However, studies have shown that multiple CVDs have largely shared genetic basis [44, 45]. Indeed, in the validation study where we tested the association of the computed CVD PRS with the CVD phenotype, the PRS was indeed positively associated with the CVD phenotype (odds ratios: 1.21–1.23). Fourth, despite of the demonstrated high consistency (93.29%) of cancer patients identified by inpatient hospital data and cancer register, the absence of data from primary and outpatient care might lead to the incomplete case identification during the recent years (i.e. cancer register can take up to 5 years to reach full coverage). Also, data on cancer treatment deemed to be limited as only those required hospital admission could be identified in our study. Fifth, as many confounders, such as lifestyle factors, were only measured at baseline, misclassification due to lack of repeated measurements for all participants might exist. However, analyses restricting to individuals with the index date right after the baseline assessment gained largely comparable results, indicating limited effect of these factors on the studied associations. Finally, the UK Biobank is not representative of the general population [46], therefore, generalisation might be a concern. Nevertheless, the close agreement between risk factor associations identified in UK Biobank data and corresponding results from nationally representative cohort studies have demonstrated sufficient generalisability [47].

In conclusion, this large community-based cohort in the UK Biobank indicated that patients with a recent cancer diagnosis were at an increased risk of multiple types of CVD. The excess CVD risk seemed to be more pronounced among individuals with low diseases susceptibilities to CVD, but generally existed across all susceptibility groups, highlighting a general necessity of enhanced psychological assistance and clinical surveillance for CVD events among all newly diagnosed cancer patients.

## Supplementary information

Supplementary tables

Supplementary figures

## Data Availability

Data from the UK Biobank (http://www.ukbiobank.ac.uk/) are available to all researchers upon making an application.
