# Supplementary material for: The role of genetic predisposition in cardiovascular risk after cancer diagnosis: a matched cohort study of the UK Biobank
Source: Br J Cancer. 2022 Aug 24;127(9):1650–9. doi: 10.1038/s41416-022-01935-y (PMC9596421; doi:10.1038/s41416-022-01935-y)
Supplement: Supplementary file 1 — Supplementary tables [file 41416_2022_1935_MOESM1_ESM.docx]

**Supplementary Table 1** **International Classification of Diseases (ICD) codes for diagnoses used in this study**

|  |  | **ICD-10** |
| --- | --- | --- |
| **Any cancer** | - | C00-C97 |
| Prostate Cancer | - | C61 |
| Breast Cancer | *-* | C50 |
| Colorectal Cancer | - | C18-C21 |
| Skin Cancer | - | C43-C44 |
| Lymphatic or hematopoietic Cancer | - | C81-C96 |
| Lung Cancer | - | C33-C34 |
| Severe Cancer | - | C15, C22, C25 |
| Other cancer |  | C00-C14, C16-C17, C23-C24, C26-C32, C35-C42, C45-C49, C51-C60, C62-C80, C97 |
| **Any cardiovascular disease** | - | I00-I09, I17-I70, I730, I74-I75 (excl. I674) |
| *Major category* | *-* |  |
| Ischemic heart disease | - | I20-I25 (excl. I25.5) |
| Cerebrovascular disease | - | I60-I69 |
| Emboli/thrombosis | - | I26, I74, I75 |
| Heart failure | - | I25.5, I42.0, I42.8, I42.9, I50 |
| Arrhythmia/conduction disorder | - | I44-I49 |
| **Acute cardiovascular events** |  | I21, I23, I24, I46, I60, I61, I63 |
| Acute myocardial infarction |  | I21, I23, I24 |
| Acute cerebrovascular disease |  | I60, I61, I63 |
| Cardiac arrest |  | I46 |
| **Covariates:** |  |  |
| History of somatic disease | Chronic pulmonary disease | J40-J47 |
|  | Connective tissue disease | M05, M06, M32-M34, M35.1, M35.3 |
|  | Diabetes | E10-E14 |
|  | Renal disease | N01, N03, N05.2-N05.7 |
|  | Liver disease | K70.2-K70.4, K71.7, K72.1, K72.9, K73, K74, K76.6, K76.7 |
|  | Ulcer disease | K25-K28 |
|  | HIV infection/AIDS | B20-B24 |
|  | Hypertension | I11-I16, I674 |
| History of psychiatric disorders | **-** | F00-F99 |
| **Chemoradiotherapy** | **-** | T66, Y842, Z08, Z510-Z512, Z541, Z542, Z926 |

**Supplementary Table 2 Top 100 GWAS variants (from Nikpay et al**^a^**) used for computing**

**polygenic risk score for cardiovascular disease (CVD)**

| SNP | CHR | BP | A1 | A2 | Beta | Se | *p* |
| --- | --- | --- | --- | --- | --- | --- | --- |
| rs2891168 | 9 | 22098619 | A | G | -0.193401 | 0.0091877 | 2.29E-98 |
| rs4977574 | 9 | 22098574 | A | G | -0.192934 | 0.0091867 | 6.35E-98 |
| rs10738610 | 9 | 22123766 | A | C | -0.19367 | 0.009292 | 1.78E-96 |
| rs10757275 | 9 | 22106225 | G | A | -0.193037 | 0.0092692 | 2.54E-96 |
| rs10757279 | 9 | 22124630 | A | G | -0.192958 | 0.0093789 | 4.73E-94 |
| rs10757277 | 9 | 22124450 | A | G | -0.192919 | 0.0093811 | 5.69E-94 |
| rs1333048 | 9 | 22125347 | A | C | -0.191445 | 0.0093105 | 5.97E-94 |
| rs10757278 | 9 | 22124477 | A | G | -0.192621 | 0.0093811 | 1.09E-93 |
| rs10757271 | 9 | 22076795 | G | A | 0.180856 | 0.009468 | 2.44E-81 |
| rs10811650 | 9 | 22067593 | A | G | -0.153987 | 0.0092366 | 2.12E-62 |
| rs7028268 | 9 | 22048414 | G | A | -0.137562 | 0.0094474 | 4.98E-48 |
| rs3217992 | 9 | 22003223 | C | T | -0.128317 | 0.009367 | 1.03E-42 |
| rs9349379 | 6 | 12903957 | A | G | -0.131836 | 0.0096527 | 1.81E-42 |
| rs10738604 | 9 | 22025493 | G | A | -0.125862 | 0.0095028 | 4.84E-40 |
| rs1537373 | 9 | 22103341 | G | T | 0.210038 | 0.0159759 | 1.77E-39 |
| rs1333042 | 9 | 22103813 | G | A | 0.209298 | 0.0159553 | 2.60E-39 |
| rs7859362 | 9 | 22105927 | C | T | 0.207873 | 0.0158477 | 2.63E-39 |
| rs1537371 | 9 | 22099568 | A | C | 0.208587 | 0.0159585 | 4.85E-39 |
| rs55730499 | 6 | 161005610 | C | T | -0.316641 | 0.0242403 | 5.39E-39 |
| rs10455872 | 6 | 161010118 | A | G | -0.318598 | 0.0243989 | 5.73E-39 |
| rs10757274 | 9 | 22096055 | A | G | -0.200768 | 0.0155088 | 2.49E-38 |
| rs7859727 | 9 | 22102165 | T | C | 0.206386 | 0.0159607 | 3.01E-38 |
| rs10738607 | 9 | 22088094 | A | G | -0.200924 | 0.0155772 | 4.59E-38 |
| rs10738608 | 9 | 22094796 | C | A | 0.208958 | 0.0163746 | 2.70E-37 |
| rs10757272 | 9 | 22088260 | C | T | -0.200517 | 0.0157402 | 3.58E-37 |
| rs118039278 | 6 | 160985526 | G | A | -0.305337 | 0.0239981 | 4.38E-37 |
| rs1537375 | 9 | 22116071 | C | T | 0.201411 | 0.0159216 | 1.12E-36 |
| rs1412829 | 9 | 22043926 | A | G | 0.122137 | 0.0098384 | 2.19E-35 |
| rs4977757 | 9 | 22094330 | G | A | 0.203806 | 0.0164191 | 2.23E-35 |
| rs10511701 | 9 | 22112599 | C | T | 0.200782 | 0.0162029 | 2.90E-35 |
| rs634537 | 9 | 22032152 | T | G | 0.121111 | 0.009821 | 6.11E-35 |
| rs679038 | 9 | 22029080 | G | A | 0.121085 | 0.00982 | 6.21E-35 |
| rs599452 | 9 | 22027402 | G | A | 0.121046 | 0.0098232 | 6.86E-35 |
| rs613312 | 9 | 22026594 | G | A | 0.121005 | 0.0098265 | 7.60E-35 |
| rs564398 | 9 | 22029547 | T | C | 0.120701 | 0.0098156 | 9.42E-35 |
| rs10811652 | 9 | 22077085 | C | A | 0.194466 | 0.0158749 | 1.68E-34 |
| rs1537370 | 9 | 22084310 | T | C | 0.1903 | 0.0156294 | 4.18E-34 |
| rs9644862 | 9 | 22090936 | G | T | 0.207289 | 0.0170872 | 7.21E-34 |
| rs62560774 | 9 | 22028406 | C | A | 0.125933 | 0.0104554 | 2.07E-33 |
| rs1333036 | 9 | 22043819 | C | T | 0.110307 | 0.0092464 | 8.28E-33 |
| rs10811656 | 9 | 22124472 | C | T | -0.20057 | 0.0168308 | 9.67E-33 |
| rs10115049 | 9 | 22032119 | G | A | 0.109647 | 0.0092149 | 1.2E-32 |
| rs10965215 | 9 | 22029445 | A | G | 0.109519 | 0.0092073 | 1.26E-32 |
| rs6475606 | 9 | 22081850 | T | C | 0.189601 | 0.0159607 | 1.52E-32 |
| rs10116277 | 9 | 22081397 | T | G | 0.189811 | 0.0159813 | 1.56E-32 |
| rs2151280 | 9 | 22034719 | A | G | 0.10975 | 0.0092421 | 1.6E-32 |
| rs4977753 | 9 | 22030027 | T | C | 0.108905 | 0.0092182 | 3.3E-32 |
| rs9644860 | 9 | 22090603 | T | C | 0.20278 | 0.0172175 | 5.1E-32 |
| rs7049105 | 9 | 22028801 | G | A | 0.10822 | 0.0092138 | 7.46E-32 |
| rs10811653 | 9 | 22091069 | T | C | 0.202914 | 0.0173305 | 1.15E-31 |
| rs2210538 | 9 | 22092257 | A | G | 0.20035 | 0.0174054 | 1.16E-30 |
| rs186696265 | 6 | 161111700 | C | T | -0.550351 | 0.0481949 | 3.35E-30 |
| rs581876 | 9 | 22022376 | C | T | 0.10962 | 0.0097298 | 1.92E-29 |
| rs10757265 | 9 | 22048859 | C | T | 0.102875 | 0.0092562 | 1.07E-28 |
| rs9644859 | 9 | 22090521 | G | A | -0.202404 | 0.0182592 | 1.48E-28 |
| rs7874604 | 9 | 22054690 | C | T | 0.109668 | 0.0098971 | 1.55E-28 |
| rs7866503 | 9 | 22091924 | G | T | -0.202877 | 0.0183635 | 2.25E-28 |
| rs7027950 | 9 | 22048391 | T | C | 0.102105 | 0.0092431 | 2.28E-28 |
| rs10757266 | 9 | 22049555 | T | C | 0.10183 | 0.0092431 | 3.17E-28 |
| rs7028570 | 9 | 22048683 | A | G | 0.101738 | 0.0092442 | 3.59E-28 |
| rs1360590 | 9 | 22041443 | C | T | 0.101839 | 0.0092616 | 4E-28 |
| rs615552 | 9 | 22026077 | T | C | 0.107351 | 0.0097798 | 4.94E-28 |
| rs568447 | 9 | 22021615 | A | G | -0.10174 | 0.0092736 | 5.27E-28 |
| rs523096 | 9 | 22019129 | A | G | 0.105282 | 0.0097646 | 4.18E-27 |
| rs944800 | 9 | 22050898 | G | A | 0.112626 | 0.0104533 | 4.55E-27 |
| rs2811713 | 9 | 21999328 | G | A | 0.107374 | 0.0100155 | 8.13E-27 |
| rs10120806 | 9 | 22047945 | C | T | 0.100799 | 0.0094593 | 1.63E-26 |
| rs10811645 | 9 | 22049656 | A | G | 0.1009 | 0.0094756 | 1.77E-26 |
| rs10811643 | 9 | 22024966 | G | A | 0.098462 | 0.0092703 | 2.37E-26 |
| rs1101330 | 9 | 22015465 | C | A | 0.10665 | 0.0100774 | 3.57E-26 |
| rs2106119 | 9 | 22017550 | G | A | 0.097055 | 0.0092605 | 1.06E-25 |
| rs2106120 | 9 | 22017101 | T | G | 0.096727 | 0.0092605 | 1.54E-25 |
| rs3218012 | 9 | 21998660 | A | G | 0.097049 | 0.0092953 | 1.62E-25 |
| rs10811640 | 9 | 22013411 | T | G | 0.096549 | 0.009266 | 2.01E-25 |
| rs573687 | 9 | 22011642 | G | A | 0.105127 | 0.0101056 | 2.41E-25 |
| rs10757263 | 9 | 22013805 | T | C | 0.096081 | 0.0092692 | 3.55E-25 |
| rs496892 | 9 | 22024351 | C | T | 0.096111 | 0.0093127 | 5.69E-25 |
| rs9295128 | 6 | 160751531 | G | T | -0.492954 | 0.048461 | 2.64E-24 |
| rs7528419 | 1 | 109817192 | A | G | 0.11453 | 0.011482 | 1.97E-23 |
| rs643319 | 9 | 22017836 | C | A | 0.092655 | 0.0093061 | 2.37E-23 |
| rs12740374 | 1 | 109817590 | G | T | 0.113549 | 0.011482 | 4.63E-23 |
| rs545226 | 9 | 22012422 | A | G | -0.091856 | 0.009367 | 1.06E-22 |
| rs597816 | 9 | 22021172 | T | C | 0.095514 | 0.0099514 | 8.15E-22 |
| rs9457927 | 6 | 160910282 | A | G | -0.357354 | 0.0373549 | 1.11E-21 |
| rs4714955 | 6 | 12903435 | C | T | 0.098544 | 0.0103935 | 2.51E-21 |
| rs140570886 | 6 | 161013013 | T | C | -0.358095 | 0.0378144 | 2.8E-21 |
| rs1101329 | 9 | 22015997 | C | T | 0.093549 | 0.009947 | 5.22E-21 |
| rs62386818 | 6 | 12923767 | C | A | 0.096274 | 0.0102914 | 8.38E-21 |
| rs62389955 | 6 | 12902248 | A | G | 0.097075 | 0.0103772 | 8.39E-21 |
| rs34343839 | 6 | 12922734 | C | T | 0.095922 | 0.0103066 | 1.32E-20 |
| rs12530250 | 6 | 12911965 | A | G | 0.096448 | 0.0103805 | 1.52E-20 |
| rs7739181 | 6 | 12934687 | G | A | 0.096854 | 0.0104902 | 2.64E-20 |
| rs7454157 | 6 | 12909874 | G | A | 0.090825 | 0.0098634 | 3.31E-20 |
| rs10757264 | 9 | 22019732 | G | A | 0.085077 | 0.0093224 | 7.1E-20 |
| rs490005 | 9 | 22020493 | G | A | 0.084218 | 0.0093192 | 1.61E-19 |
| rs629301 | 1 | 109818306 | T | G | 0.101444 | 0.0114233 | 6.66E-19 |
| rs9369640 | 6 | 12901441 | A | C | 0.089106 | 0.0100535 | 7.78E-19 |
| rs660240 | 1 | 109817838 | C | T | 0.102043 | 0.0115287 | 8.66E-19 |
| rs646776 | 1 | 109818530 | T | C | 0.101049 | 0.0114222 | 9.01E-19 |
| rs7751826 | 6 | 12900977 | T | C | 0.088127 | 0.0100741 | 2.17E-18 |

^a^ Nikpay M, Goel A, Won H-H, et al. A comprehensive 1000 Genomes–based genome-wide association meta-analysis of coronary artery disease. Nature genetics 2015; 47(10): 1121.

SNP=Single-Nucleotide Polymorphism; CHR=Chromosome; BP=Base position; A1=Risk allele; A2=Reference allele; Beta=Effect size; Se=Standard error.

**Supplementary Table 3 Characteristics of cancer patients identified by different data sources**

|  | **Cancer patients identified by both data sources** | **Cancer patients identified only by cancer register** | **Cancer patients identified only by hospital admission** |
| --- | --- | --- | --- |
|  | **(*N*=59,344)** | **(*N*=14,228)** | **(*N*=5,288)** |
| **Birth year, mean (SD)** | 1950 (7.03) | 1950 (7.22) | 1950 (7.39) |
| **Follow up time, mean (SD), y** | 7.56 (6.02) | 8.15 (6.11) | 7.01 (6.16) |
| **Age at the index date, mean (SD), y** | 62.0 (8.73) | 61.9 (9.25) | 62.3 (9.57) |
| **Sex, No.** |  |  |  |
| Female | 32095 (54.08%) | 7427 (52.20%) | 2956 (55.90%) |
| Male | 27249 (45.92%) | 6801 (47.80%) | 2332 (44.10%) |
| **Race/ethnicity, No.** |  |  |  |
| White | 57333 (96.61%) | 13916 (97.81%) | 5048 (95.46%) |
| Others | 2011 (3.39%) | 312 (2.19%) | 240 (4.54%) |
| **Townsend deprivation index, mean (SD)** | -1.50 (2.99) | -1.91 (2.78) | -1.22 (3.15) |
| **College or University degree, No.** |  |  |  |
| Yes | 17932 (30.22%) | 4857 (34.14%) | 1750 (33.09%) |
| No | 28398 (47.85%) | 6868 (48.27%) | 2423 (45.82%) |
| Unknown | 13014 (21.93%) | 2503 (17.59%) | 1115 (21.09%) |
| **Body mass index, No., kg/m^2^** |  |  |  |
| <18.5 | 305 (0.51%) | 68 (0.48%) | 25 (0.47%) |
| 18.5-24.9 | 18912 (31.87%) | 5112 (35.93%) | 1768 (33.43%) |
| 25.0-29.9 | 25810 (43.49%) | 6235 (43.82%) | 2213 (41.85%) |
| ≥30.0 | 14045 (23.67%) | 2757 (19.38%) | 1259 (23.81%) |
| Unknown | 272 (0.46%) | 56 (0.39%) | 23 (0.43%) |
| **Smoking status, No.** |  |  |  |
| Never | 30237 (50.95%) | 7858 (55.23%) | 2691 (50.89%) |
| Ever | 28764 (48.47%) | 6306 (44.32%) | 2573 (48.66%) |
| Unknown | 343 (0.58%) | 64 (0.45%) | 24 (0.45%) |
| **Smoking status, No. (%)** |  |  |  |
| Never | 2279 (3.84%) | 404 (2.84%) | 222 (4.20%) |
| Ever | 56910 (95.90%) | 13802 (97.01%) | 5046 (95.42%) |
| Unknown | 155 (0.26%) | 22 (0.15%) | 20 (0.38%) |
| **Diet types, No. (%)** |  |  |  |
| Vegetarians | 806 (1.36%) | 169 (1.19%) | 96 (1.82%) |
| Fish eaters | 1292 (2.18%) | 291 (2.05%) | 119 (2.25%) |
| Fish and poultry eaters | 1462 (2.46%) | 332 (2.33%) | 138 (2.61%) |
| Meat-eaters | 55711 (93.88%) | 13421 (94.33%) | 4928 (93.19%) |
| Unknown | 73 (0.12%) | 15 (0.11%) | 7 (0.13%) |
| **Physical activity, No. (%)** |  |  |  |
| Low | 8907 (15.01%) | 2080 (14.62%) | 780 (14.75%) |
| Moderate | 19529 (32.91%) | 4792 (33.68%) | 1776 (33.59%) |
| High | 18799 (31.68%) | 4762 (33.47%) | 1643 (31.07%) |
| Unknown | 12109 (20.40%) | 2594 (18.23%) | 1089 (20.59%) |

**Supplementary Table 4 Risk of cardiovascular disease (CVD) among patients with different cancer diagnosis, compared with their matched unexposed individuals, by different genetic risk of CVD**

| **Subtypes of cancers** | **≤6 months of follow-up** | | ***P_difference_***^d^ |  | **>6 months of follow-up** | | ***P_difference_***^d^ |
| --- | --- | --- | --- | --- | --- | --- | --- |
|  | **No. of CVD (incidence rate^c^) in cancer patients / matched unexposed individuals** | **Hazard Ratio (95% confidence interval)** |  |  | **No. of CVD (incidence rate^c^) in cancer patients / matched unexposed individuals** | **Hazard Ratio (95% confidence interval)** |  |
| **Prostate Cancer (*N*=9896)** | **208 (43.27)/522 (21.68)** | **1.97 (1.67-2.32)** |  |  | **1687 (28.02)/7346 (25.61)** | **1.05 (1.00-1.11)** |  |
| By CVD polygenic risk score^a^ |  |  | 0.038 |  |  |  | 0.028 |
| Low | 59 (46.62)/111 (17.27) | 2.64 (1.92-3.62) |  |  | 436 (27.24)/1821 (23.32) | 1.20 (1.03-1.41) |  |
| Intermediate | 52 (41.16)/141 (22.22) | 1.81 (1.32-2.49) |  |  | 461 (28.77)/2039 (26.97) | 0.99 (0.85-1.15) |  |
| High | 59 (48.18)/174 (28.10) | 1.67 (1.24-2.24) |  |  | 513 (32.94)/2263 (30.94) | 0.94 (0.81-1.09) |  |
| By CVD family history^b^ |  |  | 0.92 |  |  |  | 0.40 |
| No | 80 (38.22)/181 (17.68) | 2.10 (1.61-2.74) |  |  | 688 (26.09)/2841 (23.35) | 1.06 (0.95-1.19) |  |
| Yes | 128 (47.18)/341 (24.64) | 2.06 (1.62-2.62) |  |  | 999 (29.53)/4505 (27.28) | 1.00 (0.92-1.09) |  |
| **Breast Cancer (*N*=14561)** | **254 (35.59)/290 (8.07)** | **4.54 (3.80-5.42)** |  |  | **1761 (13.00)/6895 (10.40)** | **1.22 (1.15-1.28)** |  |
| By CVD polygenic risk score^a^ |  |  | 0.058 |  |  |  | 0.83 |
| Low | 66 (38.27)/60 (6.83) | 5.55 (3.91-7.87) |  |  | 401 (12.36)/1598 (9.87) | 1.16 (0.98-1.38) |  |
| Intermediate | 63 (36.39)/79 (8.91) | 4.02 (2.89-5.60) |  |  | 454 (13.84)/1815 (11.05) | 1.23 (1.05-1.43) |  |
| High | 65 (36.47)/94 (10.27) | 3.52 (2.57-4.83) |  |  | 536 (15.78)/2165 (12.82) | 1.19 (1.03-1.37) |  |
| By CVD family history^b^ |  |  | 0.032 |  |  |  | 0.015 |
| No | 94 (32.43)/81 (5.73) | 7.17 (4.35-11.81) |  |  | 616 (11.45)/2191 (8.49) | 1.37 (1.21-1.54) |  |
| Yes | 160 (37.75)/209 (9.58) | 3.89 (3.03-4.99) |  |  | 1145 (14.03)/4704 (11.62) | 1.15 (1.07-1.24) |  |
| **Colorectal Cancer (*N*=5905)** | **340 (124.09)/221 (15.35)** | **8.11 (6.76-9.73)** |  |  | **930 (25.09)/3659 (17.89)** | **1.38 (1.27-1.49)** |  |
| By CVD polygenic risk score^a^ |  |  | 0.44 |  |  |  | 0.66 |
| Low | 84 (113.43)/48 (13.17) | 8.38 (5.88-11.95) |  |  | 225 (22.07)/873 (16.43) | 1.44 (1.13-1.82) |  |
| Intermediate | 99 (137.37)/61 (16.62) | 8.05 (5.85-11.08) |  |  | 258 (26.95)/975 (18.81) | 1.66 (1.33-2.07) |  |
| High | 100 (152.12)/78 (20.85) | 6.99 (5.20-9.40) |  |  | 262 (28.94)/1141 (21.67) | 1.55 (1.24-1.93) |  |
| By CVD family history^b^ |  |  | 0.19 |  |  |  | 0.072 |
| No | 147 (125.25)/79 (13.61) | 8.85 (6.73-11.64) |  |  | 360 (23.29)/1292 (15.52) | 1.60 (1.36-1.87) |  |
| Yes | 193 (123.21)/142 (16.52) | 6.83 (5.19-9.00) |  |  | 570 (26.39)/2367 (19.52) | 1.34 (1.20-1.50) |  |
| **Skin Cancer *(N*=27133)** | **231 (17.37)/980 (14.78)** | **1.18 (1.02-1.37)** |  |  | **3526 (17.12)/16149 (16.83)** | **1.00 (0.97-1.04)** |  |
| By CVD polygenic risk score^a^ |  |  | 0.18 |  |  |  | 0.23 |
| Low | 59 (15.76)/207 (12.34) | 1.24 (0.93-1.66) |  |  | 949 (16.20)/3720 (15.30) | 1.06 (0.95-1.18) |  |
| Intermediate | 76 (21.68)/263 (15.47) | 1.37 (1.06-1.77) |  |  | 951 (17.62)/4422 (18.06) | 0.96 (0.87-1.07) |  |
| High | 67 (19.45)/340 (20.15) | 0.95 (0.73-1.23) |  |  | 1102 (20.80)/5016 (20.61) | 0.97 (0.88-1.07) |  |
| By CVD family history^b^ |  |  | 0.82 |  |  |  | 0.54 |
| No | 82 (14.85)/355 (13.11) | 1.27 (0.95-1.70) |  |  | 1332 (15.54)/5765 (14.76) | 1.03 (0.95-1.11) |  |
| Yes | 149 (19.16)/625 (15.94) | 1.22 (1.00-1.49) |  |  | 2194 (18.24)/10384 (18.26) | 1.00 (0.95-1.06) |  |
| **Lymphatic or hematopoietic** | **236 (114.63)/171 (15.90)** | **7.72 (6.22-9.58)** |  |  | **915 (31.97)/2548 (16.07)** | **2.02 (1.86-2.19)** |  |
| **Cancer (*N*=4405)** |  |  |  |  |  |  |  |
| By CVD polygenic risk score^a^ |  |  | 0.00078 |  |  |  | 0.055 |
| Low | 77 (144.87)/41 (15.01) | 9.24 (6.32-13.51) |  |  | 218 (29.38)/597 (14.76) | 1.78 (1.37-2.33) |  |
| Intermediate | 60 (118.62)/47 (17.39) | 6.52 (4.45-9.57) |  |  | 211 (30.35)/691 (17.41) | 1.71 (1.32-2.22) |  |
| High | 40 (76.54)/56 (20.53) | 3.56 (2.37-5.34) |  |  | 283 (39.91)/783 (19.90) | 2.54 (1.98-3.26) |  |
| By CVD family history^b^ |  |  | 0.99 |  |  |  | 0.00159 |
| No | 108 (121.90)/57 (12.86) | 9.23 (6.69-12.73) |  |  | 368 (29.62)/872 (13.13) | 2.53 (2.13-3.02) |  |
| Yes | 128 (109.13)/114 (18.03) | 9.26 (6.32-13.58) |  |  | 547 (33.78)/1676 (18.20) | 1.80 (1.60-2.03) |  |
| **Lung Cancer (*N*=2182)** | **295 (345.57)/90 (16.97)** | **20.69 (15.06-28.41)** |  |  | **294 (61.81)/1079 (20.22)** | **2.94 (2.49-3.47)** |  |
| By CVD polygenic risk score^a^ |  |  | 0.32 |  |  |  | 0.55 |
| Low | 89 (397.96)/20 (14.83) | 25.46 (15.56-41.66) |  |  | 80 (70.57)/268 (19.89) | 3.09 (1.76-5.43) |  |
| Intermediate | 79 (378.24)/26 (19.49) | 16.76 (10.62-26.46) |  |  | 67 (59.52)/290 (21.65) | 3.11 (1.78-5.42) |  |
| High | 82 (414.43)/27 (19.60) | 18.24 (11.71-28.42) |  |  | 82 (69.49)/334 (24.39) | 2.45 (1.49-4.05) |  |
| By CVD family history^b^ |  |  | 0.55 |  |  |  | 0.93 |
| No | 124 (330.45)/37 (17.25) | 16.84 (11.56-24.54) |  |  | 122 (59.18)/415 (19.39) | 3.15 (2.21-4.47) |  |
| Yes | 171 (357.44)/53 (16.78) | 19.58 (14.32-26.77) |  |  | 172 (63.82)/664 (20.78) | 3.09 (2.40-3.97) |  |
| **Severe Cancer (*N*=1916)** | **270 (361.88)/96 (20.61)** | **16.93 (12.85-22.30)** |  |  | **220 (53.78)/991 (20.94)** | **2.74 (2.29-3.28)** |  |
| By CVD polygenic risk score^a^ |  |  | 0.096 |  |  |  | 0.21 |
| Low | 74 (385.34)/20 (16.67) | 22.28 (13.56-36.60) |  |  | 61 (58.64)/235 (19.75) | 3.87 (2.16-6.95) |  |
| Intermediate | 67 (347.71)/25 (20.80) | 16.66 (10.50-26.41) |  |  | 56 (53.20)/260 (21.05) | 2.21 (1.26-3.88) |  |
| High | 85 (447.18)/38 (32.22) | 13.07 (8.89-19.20) |  |  | 55 (57.37)/311 (26.90) | 2.33 (1.35-4.03) |  |
| By CVD family history^b^ |  |  | 0.24 |  |  |  | 0.36 |
| No | 111 (331.93)/29 (15.33) | 20.94 (13.88-31.59) |  |  | 89 (55.66)/374 (19.44) | 3.10 (2.12-4.52) |  |
| Yes | 159 (386.2)/67 (24.23) | 15.50 (11.64-20.64) |  |  | 131 (52.57)/617 (21.96) | 2.50 (1.92-3.25) |  |
| **Other Cancer (*N*=15056)** | **729 (106.41)/511 (13.87)** | **7.56 (6.70-8.54)** |  |  | **2101 (21.78)/8359 (15.21)** | **1.44 (1.36-1.51)** |  |
| By CVD polygenic risk score^a^ |  |  | 0.29 |  |  |  | 0.014 |
| Low | 207 (115.48)/111 (12.05) | 9.62 (6.09-15.20) |  |  | 548 (21.30)/1974 (14.40) | 1.51 (1.30-1.77) |  |
| Intermediate | 207 (121.16)/128 (13.66) | 8.96 (5.78-13.88) |  |  | 544 (23.22)/2183 (15.73) | 1.41 (1.21-1.64) |  |
| High | 204 (120.81)/181 (19.46) | 6.92 (4.64-10.33) |  |  | 581 (25.05)/2647 (19.18) | 1.16 (1.00-1.33) |  |
| By CVD family history^b^ |  |  | 0.00022 |  |  |  | 0.034 |
| No | 317 (109.16)/159 (10.46) | 12.81 (9.33-17.58) |  |  | 833 (20.39)/3012 (13.24) | 1.54 (1.38-1.71) |  |
| Yes | 412 (104.39)/352 (16.26) | 6.43 (5.35-7.72) |  |  | 1268 (22.8)/5347 (16.60) | 1.34 (1.25-1.45) |  |

^a^ Cox model was used to estimate hazard ratios (HRs), stratified by the matching variables (i.e., birth year and sex) and adjusted for birth year, Townsend deprivation index, educational attainment, body mass index, alcohol status, smoking status, physical activity, diet types, history of psychiatric disorders, and history of somatic disease.

^b^ Cox model was used to estimate hazard ratios (HRs), stratified by the matching variables (i.e., birth year and sex), and adjusted for birth year, race/ethnicity, Townsend deprivation index, educational attainment, body mass index, alcohol status, smoking status, physical activity, diet types, history of psychiatric disorders, and history of somatic disease.

^c^ Number of cases per 1000 person-years.

^d^ The differences in hazard ratios for CVD polygenic risk score were assessed between low and high subgroups by Wald test.

**Supplementary Table 5 Risk of cardiovascular disease (CVD) among patients with a diagnosis of cancer, compared with their matched unexposed individuals, by different characteristics**

| **Characteristics** | **<3 months of follow-up** | | | |  | **3-6 months of follow-up** | |  | **>6 months of follow-up** | |
| --- | --- | --- | --- | --- | --- | --- | --- | --- | --- | --- |
|  | **No. of CVD (incidence rate^b^) in cancer patients / matched unexposed individuals** | | | **Hazard Ratio (95% confidence interval)**^c^ |  | **No. of CVD (incidence rate^b^) in cancer patients / matched unexposed individuals** | **Hazard Ratio (95% confidence interval)**^c^ |  | **No. of CVD (incidence rate^b^) in cancer patients / matched unexposed individuals** | **Hazard Ratio (95% confidence interval)^c^** |
| **By age at index time, years** | | | |  |  |  |  |  |  |  |
| ≤58 | 188 (29.95)/172 (5.20) | | | 6.80 (5.32-8.69) |  | 112 (18.12)/161 (4.91) | 4.02 (3.00-5.38) |  | 3370 (11.93)/14132 (9.63) | 1.30 (1.24-1.35) |
| 59-65 | 389 (61.36)/425 (12.97) | | | 4.83 (4.10-5.70) |  | 251 (40.96)/409 (12.69) | 3.65 (3.02-4.42) |  | 4369 (22.89)/18286 (19.39) | 1.18 (1.14-1.23) |
| ≥66 | 935 (141.08)/819 (25.74) | | | 5.39 (4.88-5.96) |  | 495 (81.28)/810 (26.69) | 3.07 (2.71-3.46) |  | 3493 (38.37)/13515 (30.21) | 1.30 (1.25-1.36) |
| *P_difference_* |  | | | 0.20 |  |  | 0.33 |  |  | 0.00082 |
| **By sex** |  | | |  |  |  |  |  |  |  |
| Female | 707 (67.99)/513 (9.74) | | | 7.03 (6.24-7.92) |  | 346 (34.60)/495 (9.59) | 3.63 (3.14-4.20) |  | 4818 (14.43)/19265 (11.46) | 1.24 (1.20-1.28) |
| Male | 805 (90.99)/903 (20.09) | | | 4.36 (3.95-4.81) |  | 512 (60.96)/885 (20.23) | 2.92 (2.61-3.27) |  | 6414 (27.83)/26668 (22.67) | 1.20 (1.16-1.23) |
| *P_difference_* |  | | | <0.0001 |  |  | 0.021 |  |  | 0.14 |
| **By history of psychiatric disorders** | | |  |  |  |  |  |  |  |  |
| No | 1166 (72.64)/1108 (13.46) | | | 5.12 (4.68-5.60) |  | 655 (42.50)/1056 (13.12) | 3.23 (2.91-3.59) |  | 9436 (19.12)/38300 (15.39) | 1.21 (1.19-1.24) |
| Yes | 346 (108.33)/308 (20.10) | | | 5.82 (4.26-7.95) |  | 203 (67.95)/324 (21.82) | 3.59 (2.49-5.17) |  | 1796 (25.41)/7633 (20.65) | 1.31 (1.19-1.45) |
| *P_difference_* |  | | | 0.44 |  |  | 0.59 |  |  | 0.13 |
| **By history of comorbidities^a^** | |  | |  |  |  |  |  |  |  |
| No | 1028 (69.11)/963 (12.53) | | | 5.40 (4.90-5.97) |  | 560 (39.14)/931 (12.38) | 3.26 (2.90-3.67) |  | 8319 (18.02)/33716 (14.38) | 1.23 (1.20-1.26) |
| Yes | 484 (110.75)/453 (21.81) | | | 4.94 (3.98-6.13) |  | 298 (72.89)/449 (22.27) | 3.09 (2.43-3.94) |  | 2913 (28.4)/12217 (23.79) | 1.19 (1.11-1.27) |
| *P_difference_* |  | | | 0.46 |  |  | 0.70 |  |  | 0.37 |

^a^ History of chronic pulmonary disease, connective tissue disease, diabetes, renal disease, liver disease, ulcer disease, HIV infection/AIDS, and hypertension.

^b^ Number of cases per 1000 person-years.

^c^ Cox model was used to estimate hazard ratios (HRs), stratified by the matching variables (i.e., birth year and sex), and adjusted for birth year, race/ethnicity, Townsend deprivation index, educational attainment, body mass index, alcohol status, smoking status, physical activity, diet types, history of psychiatric disorders, history of somatic disease, and family history of CVD.

**Supplementary Table 6 Association between cancer diagnosis and cardiovascular disease (CVD), sub-grouped by the status of chemoradiotherapy**

| **By chemoradiotherapy** | **<3 months of follow-up** | |  | **3-6 months of follow-up** | |  | **>6 months of follow-up** | |
| --- | --- | --- | --- | --- | --- | --- | --- | --- |
|  | **No. of CVD (incidence rate**^a^**) in cancer patients / matched unexposed individuals** | **Hazard Ratio**^b^ **(95% confidence interval)** |  | **No. of CVD (incidence rate**^a^**) in cancer patients / matched unexposed individuals** | **Hazard Ratio**^b^ **(95% confidence interval)** |  | **No. of CVD (incidence rate**^a^**) in cancer patients / matched unexposed individuals** | **Hazard Ratio**^b^ **(95% confidence interval)** |
| *Yes* | 657 (117.06)/1416 (8.76) | 6.11 (5.00-7.46) |  | 488 (90.42)/1380 (5.12) | 5.15 (4.16-6.37) |  | 4657 (27.62)/45933 (1.63) | 1.14 (1.09-1.19) |
| *No* | 855 (62.72)/1416 (8.76) | 4.30 (3.95-4.69) |  | 370 (28.46)/1380 (5.12) | 1.95 (1.74-2.19) |  | 6575 (16.62)/45933 (1.63) | 1.03 (1.00-1.06) |
| *P_difference_* |  | 0.0016 |  |  | < 0.0001 |  |  | 0.0016 |

^a^ Number of cases per 1000 person-years.

^b^ Cox model was used to estimate hazard ratios (HRs), stratified by the matching variables (i.e., birth year and sex), and adjusted for birth year, race/ethnicity, Townsend deprivation index, educational attainment, body mass index, alcohol status, smoking status, physical activity, diet types, history of psychiatric disorders, history of somatic disease, and family history of CVD.

**Supplementary Table 7 Risk of cardiovascular disease (CVD) among individuals with a diagnosis of cancer, stratified by the polygenic risk score (PRS) of anxiety or stress-related disorders (SRD)**

| **Level of PRS** | **<3 months of follow-up** | |  | **3-6 months of follow-up** | |  | **>6 months of follow-up** | |
| --- | --- | --- | --- | --- | --- | --- | --- | --- |
|  | **No. of CVD (incidence rate**^b^**) in cancer patients / matched unexposed individuals** | **Hazard Ratio^c^ (95% confidence interval)** |  | **No. of CVD in patients (incidence**^b^**)/ matched unexposed individuals** | **Hazard Ratio^c^ (95% confidence interval)** |  | **No. of CVD in patients (incidence**^b^**)/ matched unexposed individuals** | **Hazard Ratio^c^ (95% confidence interval)** |
| *Low* | 379 (76.30)/392 (15.79) | 4.28 (3.37-5.43) |  | 219 (46.06)/333 (13.74) | 3.26 (2.45-4.34) |  | 2916 (20.18)/11918 (16.44) | 1.19 (1.12-1.27) |
| *Intermediate* | 396 (79.45)/405 (16.33) | 5.72 (4.43-7.39) |  | 253 (53.12)/421 (17.40) | 3.41 (2.62-4.44) |  | 3039 (20.77)/12613 (17.42) | 1.16 (1.10-1.24) |
| *High* | 460 (93.24)/392 (15.77) | 6.18 (4.87-7.85) |  | 245 (52.14)/361 (14.86) | 3.69 (2.81-4.86) |  | 3208 (22.26)/13000 (17.96) | 1.17 (1.10-1.24) |
| *P_difference_^a^* |  | 0.033 |  |  | 0.54 |  |  | 0.70 |

^a^ The differences in hazard ratios for anxiety or SRD PRS were assessed between low and high subgroups by Wald test.

^b^ Number of cases per 1000 person-years.

^c^ Cox model was used to estimate hazard ratios (HRs), stratified by the matching variables (i.e., birth year and sex), and adjusted for birth year, race/ethnicity, Townsend deprivation index, educational attainment, body mass index, alcohol status, smoking status, physical activity, diet types, history of psychiatric disorders, history of somatic disease, and family history of CVD.

**Supplementary Table 8 Sensitive analysis of risk of cardiovascular disease (CVD), identified according to the primary diagnosis/underlying death cause, among patients with a diagnosis of cancer, compared with their matched unexposed individuals**

| **Characteristics** | **<3 months of follow-up** | |  | **3-6 months of follow-up** | |  | **>6 months of follow-up** | |
| --- | --- | --- | --- | --- | --- | --- | --- | --- |
|  | **No. of CVD (incidence rate**^e^**) in cancer patients / matched unexposed individuals** | **Hazard Ratio (95% confidence interval)** |  | **No. of CVD (incidence rate**^e^**) in cancer patients / matched unexposed individuals** | **Hazard Ratio (95% confidence interval)** |  | **No. of CVD (incidence rate**^e^**) in cancer patients / matched unexposed individuals** | **Hazard Ratio (95% confidence interval)** |
| **Overall***^a^* | 323 (16.66)/642 (6.57) | 2.46 (2.14-2.82) |  | 308 (16.47)/602 (6.30) | 2.52 (2.19-2.91) |  | 4497 (7.59)/19497 (6.59) | 1.14 (1.10-1.18) |
| ***By CVD polygenic risk score, by tertiles****^b^* |  |  |  |  |  |  |  |  |
| Low | 87 (16.89)/140 (5.67) | 3.18 (2.05-4.94) |  | 82 (16.53)/127 (5.25) | 3.03 (1.95-4.70) |  | 1092 (6.93)/4377 (5.86) | 1.16 (1.05-1.28) |
| Intermediate | 90 (18.14)/174 (7.00) | 3.05 (2.00-4.65) |  | 84 (17.55)/158 (6.50) | 2.33 (1.53-3.55) |  | 1179 (7.84)/5240 (6.98) | 1.07 (0.97-1.17) |
| High | 91 (18.58)/227 (9.10) | 1.66 (1.13-2.43) |  | 100 (21.19)/213 (8.73) | 1.69 (1.16-2.47) |  | 1419 (9.45)/6404 (8.46) | 1.08 (0.99-1.18) |
| *P_difference_*^c^ |  | 0.029 |  |  | 0.048 |  |  | 0.28 |
| ***By CVD family history***^d^ |  |  |  |  |  |  |  |  |
| No | 131 (16.06)/208 (5.22) | 2.91 (2.18-3.88) |  | 114 (14.51)/199 (5.10) | 3.13 (2.26-4.34) |  | 1705 (6.93)/6600 (5.51) | 1.18 (1.10-1.26) |
| Yes | 192 (17.09)/434 (7.50) | 2.29 (1.88-2.78) |  | 194 (17.88)/403 (7.12) | 2.61 (2.13-3.21) |  | 2792 (8.05)/12897 (7.32) | 1.09 (1.04-1.14) |
| *P_difference_* |  | 0.18 |  |  | 0.36 |  |  | 0.059 |

^a^ Cox model was used to estimate hazard ratios (HRs), stratified by the matching variables (i.e., birth year and sex) and adjusted for birth year, race/ethnicity, Townsend deprivation index, educational attainment, body mass index, alcohol status, smoking status, physical activity, diet types, history of psychiatric disorders, history of somatic disease, and family history of CVD.

^b^ Cox model was used to estimate hazard ratios (HRs), stratified by the matching variables (i.e., birth year and sex), and adjusted for birth year, Townsend deprivation index, educational attainment, body mass index, alcohol status, smoking status, physical activity, diet types, history of psychiatric disorders, and history of somatic disease.

^c^ The differences in hazard ratios for CVD polygenic risk score were assessed between low and high subgroups by Wald test.

^d^ Cox model was used to estimate hazard ratios (HRs), stratified by the matching variables (i.e., birth year and sex) and adjusted for birth year, race/ethnicity, Townsend deprivation index, educational attainment, body mass index, alcohol status, smoking status, physical activity, diet types, history of psychiatric disorders, history of somatic disease.

^e^ Number of cases per 1000 person-years.

**Supplementary Table 9 Risk of cardiovascular disease (CVD) among cancer patients diagnosed within one year after baseline (*N*=4,133), compared with their matched unexposed individuals (*N*=19,045)**

| **Characteristics** | **<3 months of follow-up** | |  | **3-6 months of follow-up** | |  | **>6 months of follow-up** | |
| --- | --- | --- | --- | --- | --- | --- | --- | --- |
|  | **No. of CVD (incidence rate**^e^**) in cancer patients / matched unexposed individuals** | **Hazard Ratio (95% confidence interval)** |  | **No. of CVD (incidence rate**^e^**) in cancer patients / matched unexposed individuals** | **Hazard Ratio (95% confidence interval)** |  | **No. of CVD (incidence rate**^e^**) in cancer patients / matched unexposed individuals** | **Hazard Ratio (95% confidence interval)** |
| **Overall***^a^* | 73 (71.90)/67 (14.09) | 4.99 (3.58-6.96) |  | 46 (46.81)/53 (11.22) | 4.03 (2.71-6.00) |  | 702 (22.31)/2860 (17.97) | 1.20 (1.10-1.30) |
| ***By CVD polygenic risk score, by tertiles****^b^* |  |  |  |  |  |  |  |  |
| Low | 28 (107.65)/14 (11.60) | 9.88 (5.17-18.87) |  | 12 (47.96)/12 (10.01) | 4.50 (2.05-9.90) |  | 180 (22.43)/663 (16.33) | 1.31 (1.11-1.54) |
| Intermediate | 20 (73.53)/19 (15.35) | 4.45 (2.36-8.39) |  | 15 (57.14)/13 (10.57) | 5.46 (2.51-11.87) |  | 191 (22.74)/793 (19.30) | 1.15 (0.98-1.35) |
| High | 15 (58.40)/23 (19.07) | 3.06 (1.58-5.91) |  | 12 (48.14)/19 (15.90) | 3.14 (1.52-6.45) |  | 208 (26.35)/873 (22.16) | 1.13 (0.97-1.32) |
| *P_difference_*^c^ |  | 0.013 |  |  | 0.51 |  |  | 0.20 |
| ***By CVD family history***^d^ |  |  |  |  |  |  |  |  |
| No | 31 (72.63)/24 (12.48) | 5.73 (3.35-9.81) |  | 17 (41.04)/17 (8.90) | 4.35 (2.21-8.53) |  | 281 (21.20)/1055 (16.30) | 1.26 (1.11-1.44) |
| Yes | 42 (71.38)/43 (15.18) | 4.60 (3.00-7.06) |  | 29 (51.01)/36 (12.80) | 3.90 (2.38-6.40) |  | 421 (23.11)/1805 (19.11) | 1.16 (1.04-1.29) |
| *P_difference_* |  | 0.53 |  |  | 0.80 |  |  | 0.34 |

^a^ Cox model was used to estimate hazard ratios (HRs), stratified by the matching variables (i.e., birth year and sex) and adjusted for birth year, race/ethnicity, Townsend deprivation index, educational attainment, body mass index, alcohol status, smoking status, physical activity, diet types, history of psychiatric disorders, history of somatic disease, and family history of CVD.

^b^ Cox model was used to estimate hazard ratios (HRs), stratified by the matching variables (i.e., birth year and sex), and adjusted for birth year, Townsend deprivation index, educational attainment, body mass index, alcohol status, smoking status, physical activity, diet types, history of psychiatric disorders, and history of somatic disease.

^c^ The differences in hazard ratios for CVD polygenic risk score were assessed between low and high subgroups by Wald test.

^d^ Cox model was used to estimate hazard ratios (HRs), stratified by the matching variables (i.e., birth year and sex) and adjusted for birth year, race/ethnicity, Townsend deprivation index, educational attainment, body mass index, alcohol status, smoking status, physical activity, diet types, history of psychiatric disorders, history of somatic disease.

^e^ Number of cases per 1000 person-years.

**Supplementary Table 10 Risk of cardiovascular disease (CVD) among individuals with a diagnosis of cancer, additionally adjusting or stratifying by the number of hospital admissions during the first year of follow-up, compared with their matched unexposed individuals**

|  | **<3 months of follow-up** | |  | **3-6 months of follow-up** | |  | **>6 months of follow-up** | |
| --- | --- | --- | --- | --- | --- | --- | --- | --- |
|  | **No. of cases (incidence rate**^b^**) in cancer patients / matched unexposed individuals** | **Hazard Ratio (95% confidence interval)** |  | **No. of cases (incidence rate**^b^**) in cancer patients / matched unexposed individuals** | **Hazard Ratio (95% confidence interval)** |  | **No. of cases (incidence rate**^b^**) in cancer patients / matched unexposed individuals** | **Hazard Ratio (95% confidence interval)** |
| **Further adjusted by the number of hospital admissions during the first year of follow-up strategy** | | | | | | | | |
| Full model^a^ +number of hospital admissions during the first year of follow-up | 1512 (78.56)/1416 (14.50) | 2.98 (2.73-3.26) |  | 858 (46.64)/1380 (14.47) | 1.59 (1.42-1.78) |  | 11232 (19.90)/45933 (16.07) | 1.18 (1.15-1.21) |
| **Stratified by the number of hospital admissions during the first year of follow-up** | | | | | | | | |
| ≤2 | 260 (19.06)/763 (8.45) | 2.24 (1.92-2.61) |  | 141 (10.57)/759 (8.59) | 1.22 (1.01-1.47) |  | 7694 (17.13)/41821 (15.53) | 1.05 (1.02-1.07) |
| >2 | 1252 (223.40)/653 (88.53) | 2.46 (2.07-2.92) |  | 717 (141.67)/621 (88.96) | 1.29 (1.04-1.60) |  | 3538 (30.76)/4112 (24.82) | 1.34 (1.21-1.49) |

^a^ Cox model was used to estimate hazard ratios (HRs), stratified by the matching variables (i.e., birth year and sex) and adjusted for birth year, race/ethnicity, Townsend deprivation index, educational attainment, body mass index, alcohol status, smoking status, physical activity, diet types, history of psychiatric disorders, history of somatic disease, and family history of CVD.

^b^ Number of cases per 1000 person-years.
