# Supplementary material for: The role of genetic predisposition in cardiovascular risk after cancer diagnosis: a matched cohort study of the UK Biobank
Source: Br J Cancer. 2022 Aug 24;127(9):1650–9. doi: 10.1038/s41416-022-01935-y (PMC9596421; doi:10.1038/s41416-022-01935-y)
Supplement: Supplementary file 2 — Supplementary figures [file 41416_2022_1935_MOESM2_ESM.docx]

| 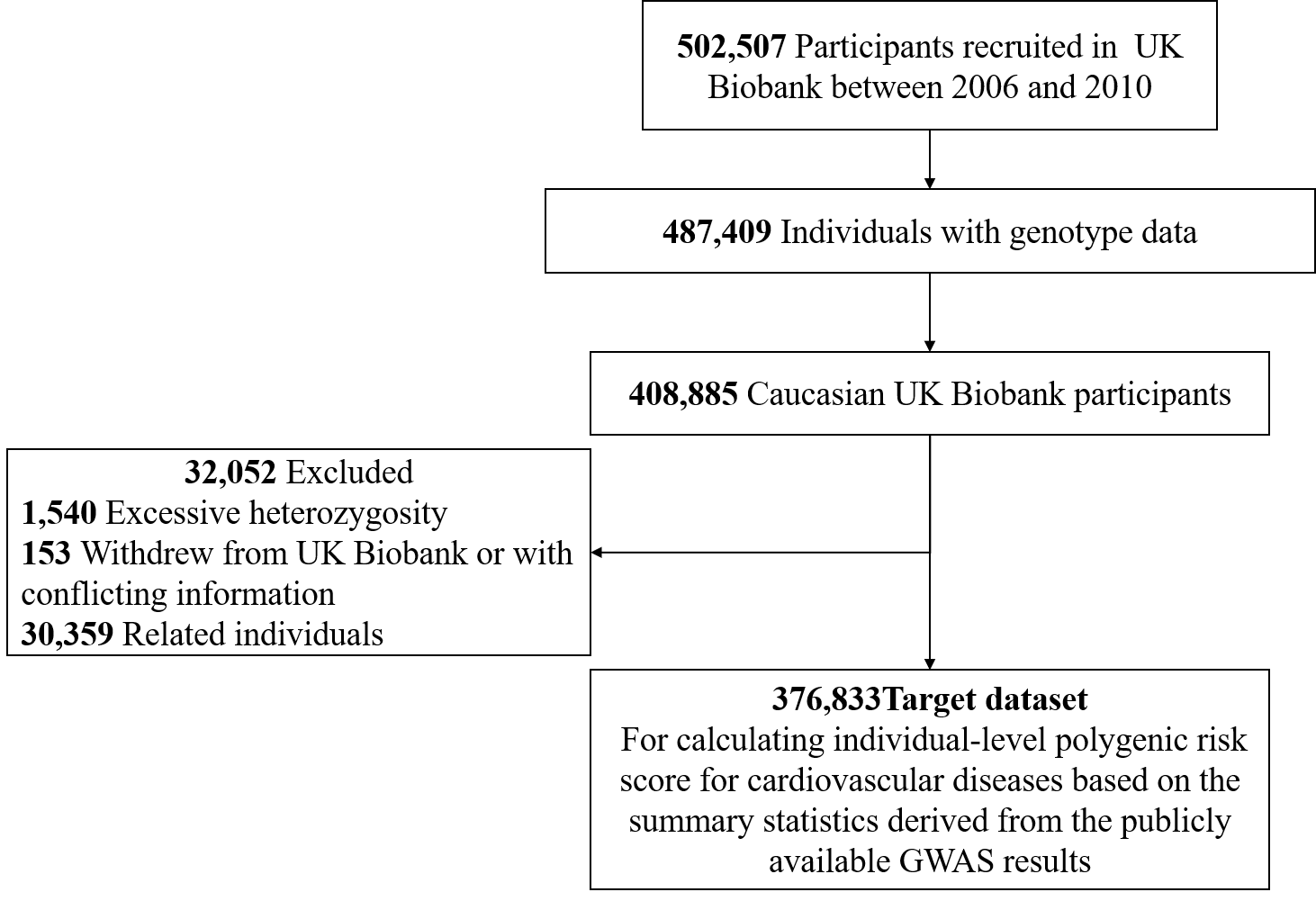 |
| --- |
| **Supplementary Figure 1** **Flow of participants for the calculation of cardiovascular disease polygenic risk score** |

| 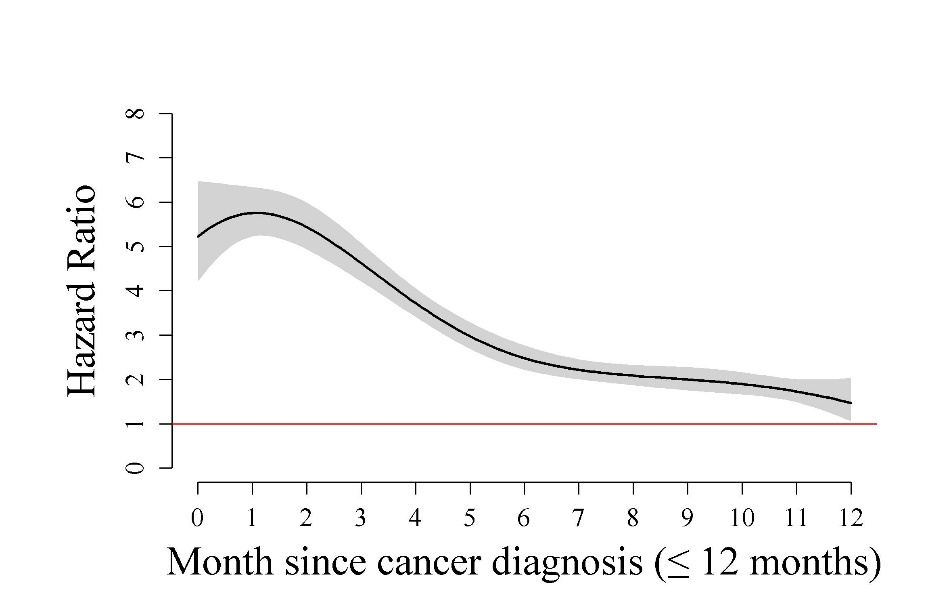 | 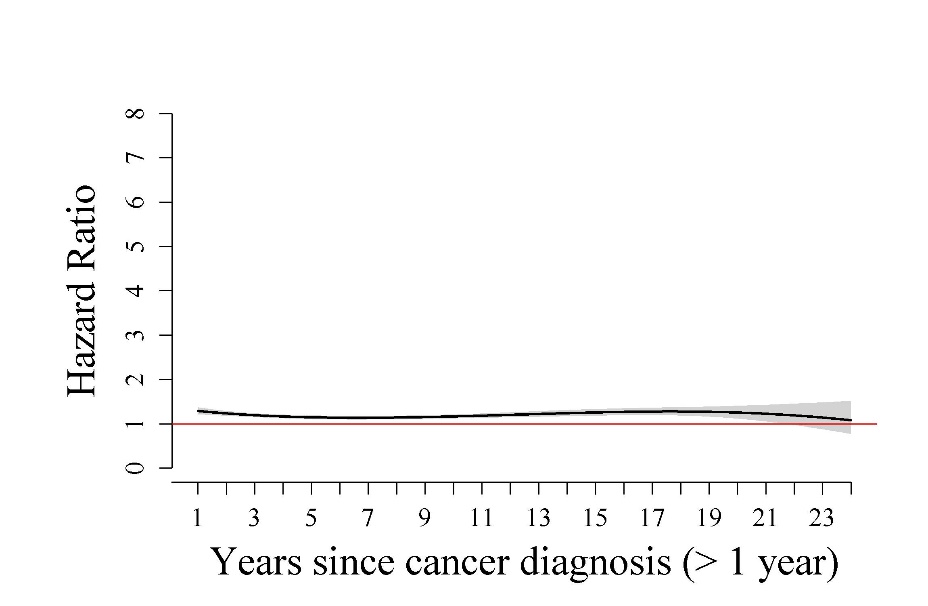 |
| --- | --- |
| **Supplementary Figure 2** **Change of the relative risk for cardiovascular disease over follow-up time among patients with a diagnosis of cancer, compared to matched unexposed individuals** | |
